# Examining the Impact of Individual and Shared Biological Risks on Health among Older Married Couples

**DOI:** 10.1093/geroni/igab046.3597

**Published:** 2021-12-17

**Authors:** Tai-Te Su, Shannon Meija, Richard Gonzalez

**Affiliations:** 1 University of Illinois at Urbana-Champaign, United States; 2 University of Illinois, Champaign, Illinois, United States; 3 University of Michigan, Ann Arbor, Michigan, United States

## Abstract

Relationship research has suggested that health among spouses is interdependent and should be considered jointly. Using data from the 2008/2010 and 2016/2018 waves of the Health and Retirement Study (3858 qualified couples; age=67.0±9.6), we investigated the joint influence of married partners’ individual and shared cumulative biological risk on future health outcomes. Two risk indicators were constructed to indicate biological health in different domains. Individual grip strength, walk speed, lung function, and cystatin-C were biomarkers selected to construct frailty risk whereas blood pressure, pulse, waist circumference, C-reactive protein, glycohemoglobin, high-density lipoprotein cholesterol, and total cholesterol were biomarkers used to construct cardiometabolic risk. Shared risk was calculated as the number of risks the partners shared. We employed multilevel Poisson regression models to nest partners within couples and examine the effects of individual and shared cumulative risks on future functional limitations. Heckman correction was performed to correct potential selection bias. Our unadjusted models showed individual (frailty: b=0.22, p<.001; cardiometabolic: b=0.10, p<.001) and shared (frailty: b=0.17, p<.001; cardiometabolic: b=0.08, p<.01) risks are associated with greater future functional limitations. Further, shared cardiometabolic risk moderated the effect of individual risk (b=-0.01, p<.05). In the adjusted models, the direct associations between shared risks and future functional limitations were explained by indicators of partner selection and shared experiences. In the fully adjusted model, the cross-level interaction for frailty risk became statistically significant. The unique set of dynamics shown in our study offered new insights into understanding how couples influence one another in the context of multisystem biological health.

